# Network structure analysis of post-traumatic stress and depressive symptoms among college students: identification of central and bridge symptoms

**DOI:** 10.3389/fpsyt.2026.1825172

**Published:** 2026-04-29

**Authors:** Yongtao Yan, Zongyu Liu, Haitao Niu

**Affiliations:** 1 School of Physical Education, Shenzhen Polytechnic University, Shenzhen, China; 2 Department of Sports Science, College of Education, Zhejiang University, Hangzhou, China; 3 School of Physical Education, Shandong University, Jinan, China

**Keywords:** bridge symptoms, college students, depression, mental health, network analysis, post-traumatic stress

## Abstract

**Objective:**

The comorbidity between post-traumatic stress disorder (PTSD) and depression has received increasing attention among college students; however, the interaction mechanisms at the symptom level remain unclear. This study employed network analysis to construct a cross-diagnostic network structure of post-traumatic stress and depressive symptoms among college students, identifying central symptoms and bridge symptoms to provide empirical evidence for precision-oriented psychological interventions.

**Methods:**

A cross-sectional survey was conducted among 501 college students from a Chinese university using the Impact of Event Scale–Revised (IES-R) and the Patient Health Questionnaire-9 (PHQ-9). A Gaussian Graphical Model (GGM) combined with graphical LASSO was used to construct the symptom network. Expected Influence (EI) was calculated to identify central symptoms, and Bridge Expected Influence (Bridge EI) was used to identify bridge symptoms connecting post-traumatic stress and depressive domains. Network accuracy and stability were examined using bootstrap procedures.

**Results:**

The symptom network revealed complex connectivity patterns among 31 symptom nodes. Central symptom analysis indicated that H2 (nervousness/exaggerated startle response, EI = 1.38), I4 (involuntary recall, EI = 1.33), and H4 (difficulty concentrating, EI = 1.09) exhibited the highest expected influence. Bridge symptom analysis showed that I8 (trauma-related dreams, Bridge EI = 0.88), H1 (irritability, Bridge EI = 0.85), and I7 (intense emotional fluctuations, Bridge EI = 0.80) were key bridges connecting the post-traumatic stress and depressive dimensions. The network demonstrated good stability, with a CS-coefficient of 0.75.

**Conclusion:**

Nervousness/exaggerated startle response and involuntary recall are central symptoms in the post-traumatic stress–depression network among college students, while trauma-related dreams, irritability, and emotional fluctuations serve as key bridge symptoms connecting the two disorders. Clinical interventions should prioritize these hub symptoms to maximize treatment efficacy and disrupt cross-diagnostic symptom connectivity.

## 1 Introduction

Post-traumatic stress disorder (PTSD) and major depressive disorder (MDD) are among the most prevalent mental health concerns in college student populations (1, 2). Epidemiological surveys indicate that in the general Chinese college student population, the prevalence of depression reaches approximately 9.8% (3). Furthermore, under specific high-stress contexts such as the COVID-19 pandemic, the psychological burden intensifies significantly, with the prevalence rate of PTSD reported to be as high as 15.5% (4). Notably, the comorbidity rate between PTSD and depression is remarkably high; a survey by Quan et al. (4) involving nearly 7,000 college students found that the comorbidity rate reached 11.5%. Furthermore, global trends highlight an increasing psychological burden; for instance, recent surveys among United States (US) college students have shown a significant upward trend in PTSD diagnoses, rising from 3.4% in 2017–2018 to 7.5% in 2021–2022 (5). These mental health problems not only affect academic performance and interpersonal relationships but are also closely associated with increased dropout rates, substance abuse, and elevated suicide risk (6, 7). Therefore, gaining a thorough understanding of the symptom-level interaction mechanisms between PTSD and depression is of considerable importance for the development of effective intervention strategies.

Traditionally, PTSD and depression have been conceptualized as independent constructs driven by their respective underlying etiologies, with researchers typically employing latent variable models for separate assessment and intervention. However, extensive empirical research has demonstrated that these two disorders exhibit a high degree of comorbidity and complex temporal interactions. While clinical reviews suggest shared vulnerabilities and overlapping mechanisms (8), longitudinal studies have further confirmed a reciprocal relationship across time. Specifically, early MDD can predict subsequent PTSD symptom clusters just as PTSD exacerbates future depressive symptoms, demonstrating a clear bidirectional pattern (9). At the symptom level, the negative cognitions and emotional alterations associated with PTSD (such as loss of interest and emotional detachment) demonstrate substantial overlap with depressive symptoms (10), and this symptom crossover further complicates the understanding of comorbidity mechanisms. Traditional categorical diagnostic approaches and intervention strategies oriented toward single disorders are insufficient to address this challenge (11).

Network analysis provides a novel theoretical perspective and analytical framework for understanding the complex relationships among psychological symptoms. In contrast to traditional latent variable models that regard symptoms as passive indicators of underlying causes, network theory conceptualizes symptoms themselves as active elements that exert mutual causal influences, with connections (edges) between symptoms representing conditional dependency relationships (12). The network theory of mental disorders proposed by Borsboom (13) posits that mental disorders are not caused by a single latent etiology but rather emerge from the mutual activation and reinforcement among symptoms, ultimately forming a self-sustaining vicious cycle network. This theoretical framework has been widely applied in psychopathological research and has spurred new methodological developments (14, 15).

In symptom network research, centrality indices serve as key tools for identifying central symptoms. Expected Influence (EI), proposed by Robinaugh et al. (16), comprehensively accounts for both the direction and weight of edges and is considered a reliable index for evaluating the overall influence of symptoms within a network. Simulation studies have demonstrated that nodes with high expected influence exert stronger effects on overall network activation in dynamic simulations, and can thus be regarded as priority intervention targets (17). Furthermore, the bridge centrality indices proposed by Jones et al. (18) enable the identification of bridge symptoms that connect different symptom clusters. Simulation studies have confirmed that interventions targeting bridge nodes identified through bridge centrality are more effective at preventing comorbidity propagation than strategies based on traditional centrality measures (18). These bridge symptoms play a critical role in the development and maintenance of psychological comorbidity, and intervening on them may effectively block the co-occurrence of symptoms across different dimensions. In recent years, several studies have applied network analysis to the domain of PTSD and depression comorbidity. Chen et al. (19), using a large community sample of snakebite victims (N = 6,837), identified specific bridge symptoms between PTSD and depression. Wei et al. (20) explored the network characteristics of anxiety, depression, and PTSD among nursing staff and identified suicidal ideation and restlessness as key bridge symptoms. However, cross-diagnostic network analyses of post-traumatic stress and depressive symptoms specifically among college students remain limited, particularly those constructing item-level networks using the IES-R and PHQ-9.

The Impact of Event Scale–Revised (IES-R) is a well-established instrument for assessing post-traumatic stress symptoms, encompassing three subdimensions: intrusion, avoidance, and hyperarousal (21). The Patient Health Questionnaire-9 (PHQ-9) is a widely used tool for screening and evaluating the severity of depression (22). The combination of these two instruments provides an ideal framework for exploring cross-diagnostic symptom interactions between post-traumatic stress and depression. Given the unique stressors faced by college students—including intense academic competition, employment pressure, and interpersonal challenges (23)—it is necessary to conduct symptom network research on post-traumatic stress and depression within this population.

Despite growing interest in PTSD–depression network comorbidity, cross-diagnostic network analyses predominantly focus on clinical or highly traumatized populations. Studies mapping the subclinical, prodromal covariance of these symptoms within general college student populations remain limited. Furthermore, many prior studies employ subscale-level rather than item-level nodes, sacrificing the clinical granularity necessary for symptom-specific intervention.

To address these gaps, the present study constructed a full item-level (31-node) cross-diagnostic network of post-traumatic stress (IES-R) and depressive symptoms (PHQ-9) in a large Chinese college student sample. By mapping the network at a subclinical baseline and validating its pathological relevance through a Network Comparison Test (NCT), this study aims to identify the early bridge symptoms that characterize comorbidity, providing novel empirical targets for early psychological prevention. Specifically, the present study aimed to: (1) construct the cross-diagnostic network structure; (2) identify central symptoms through Expected Influence (EI); (3) identify bridge symptoms connecting post-traumatic stress and depression through Bridge Expected Influence (Bridge EI); and (4) analyze the overlap patterns between central and bridge symptoms and their implications for early targeted interventions.

## 2 Methods

### 2.1 Participants

This study employed a cross-sectional design, targeting undergraduate students enrolled in universities across multiple provinces in China. Following established methodological recommendations, data were collected through an online questionnaire using a snowball sampling strategy between May and June 2022. Specifically, graduate students at each participating university were contacted and served as intermediaries to invite undergraduates to participate and to disseminate the electronic questionnaire link. To ensure informed participation and enhance response quality, an informed consent form was provided to all prospective participants prior to data collection. The form outlined the research objectives, data confidentiality assurances, and personal anonymity guarantees, while explicitly informing participants that there were no correct or incorrect responses and that each authentic response was of significant value to the study. The survey covered four provinces—Shandong, Sichuan, Hainan, and Shaanxi—and encompassed eight institutions of higher education. A total of 520 questionnaires were distributed equally across the four provinces (130 per province), and 501 valid questionnaires were ultimately collected, yielding an effective response rate of 96.3%. Demographic characteristics of the participants are presented in **Table 1**.

**Table 1:** Demographic characteristics of participants (N = 501).

Variable	Category	n (%)
Gender	Male	260 (51.9%)
	Female	241 (48.1%)
Age (M ± SD)		20.67 ± 1.98
Academic year	Freshman	160 (31.9%)
	Sophomore	93 (18.6%)
	Junior	89 (17.8%)
	Senior	159 (31.7%)
Residential area	Urban	241 (48.1%)
	Rural	260 (51.9%)
Single-parent family	Yes	62 (12.4%)
	No	439 (87.6%)

### 2.2 Instruments

#### 2.2.1 Impact of Event Scale–Revised

The IES-R was used to assess participants’ stress response levels following the recall of a recent traumatic event (21). The instructions asked participants to recall the most impactful traumatic or stressful event they had experienced recently and to respond based on their feelings during the preceding week. The scale comprises 22 items divided into three subdimensions: Intrusion (8 items: I1, I2, I3, I4, I5, I6, I7, I8), assessing the degree to which trauma-related memories involuntarily intrude into consciousness; Avoidance (8 items: A1, A2, A3, A4, A5, A6, A7, A8), assessing the tendency to deliberately or unconsciously avoid trauma-related stimuli; and Hyperarousal (6 items: H1, H2, H3, H4, H5, H6), assessing sustained heightened vigilance and excessive reactivity. Each item is rated on a 5-point Likert scale (1 = “not at all” to 5 = “extremely”), with higher scores indicating more severe symptoms. The Chinese version of this scale has demonstrated good reliability and validity in Chinese populations (24). In the present study, the Cronbach’s α coefficient for this scale was 0.95.

#### 2.2.2 Patient Health Questionnaire-9

The PHQ-9 was used to assess the severity of depressive symptoms experienced by participants over the preceding two weeks (22). The scale comprises nine items (D1–D9) corresponding to the nine core symptoms of a depressive episode as defined in the DSM-5: loss of interest (D1), depressed mood (D2), sleep problems (D3), fatigue (D4), appetite changes (D5), self-deprecation (D6), difficulty concentrating (D7), psychomotor changes (D8), and suicidal ideation (D9). Each item is rated on a 4-point Likert scale (1 = “not at all” to 4 = “nearly every day”), with higher scores indicating more severe symptoms. The Chinese version of the PHQ-9 has demonstrated a stable single-factor structure and good internal consistency among Chinese college students (25). In the present study, the Cronbach’s α coefficient for this scale was 0.92.

### 2.3 Statistical analysis

A Gaussian Graphical Model (GGM) combined with graphical LASSO regularization (EBIC tuning parameter γ = 0.5) was employed to construct a sparse symptom network, with partial correlation coefficients characterizing the conditional dependency relationships between nodes. Expected Influence (EI) was used to identify central symptoms, and Bridge Expected Influence (Bridge EI) was used to identify cross-dimensional bridge symptoms. Specifically, Bridge EI was selected over unsigned measures (such as Bridge Strength) because it accounts for the directionality (sign) of edges when computing cross-community influence. This is particularly important for our network, which contains notable negative edges (e.g., nodes A6 and A2). Based on the theoretical structure of the scales, the 31 nodes were classified into four communities: Intrusion (I1–I8), Avoidance (A1–A8), Hyperarousal (H1–H6), and Depression (D1–D9). Raw Bridge EI values were standardized using Z-scores for comparison purposes. Network stability and accuracy were examined using bootstrap procedures (1,000 resamples) from the bootnet package, including 95% confidence interval estimation for edge weights, case-dropping stability analysis for centrality indices (CS-coefficient ≥ 0.25 as acceptable), and bootstrap difference tests. Visualization employed a Fruchterman–Reingold spring layout, where edge thickness corresponded to the absolute value of partial correlations, blue and red edges represented positive and negative connections respectively, and the four dimensions were distinguished by different node colors. Furthermore, to evaluate the clinical relevance of the network structure across different symptom severity levels, a Network Comparison Test (NCT) with 1,000 permutations was conducted to compare global network strength and overall network structure between high- and low-depression subgroups. All analyses were conducted in R version 4.4.1, primarily using the bootnet, qgraph, networktools, NetworkComparisonTest and ggplot2 packages.

## 3 Results

### 3.1 Descriptive statistics

**Table 2** presents the descriptive statistics for the 31 symptom items. To evaluate the overall severity of the sample, total scale scores were examined relative to their scoring systems. The PHQ-9 items were recorded on a 1–4 scale (total range: 9–36). The mean PHQ-9 total score was 14.38 (SD = 6.16). To compare this against standard clinical thresholds (0–27 scale), the established cutoff for moderate depression (≥10, 22) was mathematically adjusted upward by the baseline to an equivalent cutoff of ≥19. Similarly, for post-traumatic stress, the IES-R was recorded on a 1–5 scale (total range: 22–110), yielding a mean of 40.99 (SD = 14.22). According to a recent validation study by Chang et al. (2024), the optimal cutoff for DSM-5 PTSD is ≥25 on the standard 0–88 scale, which is mathematically equivalent to ≥47 on our 1–5 scale (26). Both mean scores fell below these adjusted clinical screening thresholds, clearly indicating that the sample, on average, experienced mild, subclinical levels of psychological distress. This severity profile is highly consistent with a general, non-clinical college student population navigating daily academic, interpersonal, and life stressors, providing a baseline to examine early, prodromal symptom networks. Among the 22 IES-R items, A6 (emotional numbness; M = 2.34, SD = 1.17) had the highest score, while H5 (physiological reactions; M = 1.60, SD = 0.78) had the lowest. Among the nine PHQ-9 items, D4 (fatigue; M = 1.71, SD = 0.91) had the highest score, and D9 (suicidal ideation; M = 1.34, SD = 0.77) had the lowest.

**Table 2:** Descriptive statistics for the 31 symptom items (N = 501).

Node	Symptom description	Dimension	M	SD
I1	Triggered re-experiencing	Intrusion	2.24	0.97
I2	Sleep disruption	Intrusion	1.77	0.86
I3	Associative intrusion	Intrusion	2.14	0.92
I4	Involuntary recall	Intrusion	1.94	0.93
I5	Flashbacks	Intrusion	1.94	0.88
I6	Reliving the experience	Intrusion	1.84	0.91
I7	Emotional fluctuations	Intrusion	1.78	0.85
I8	Trauma-related dreams	Intrusion	1.70	0.78
A1	Avoiding distress	Avoidance	1.96	0.95
A2	Derealization	Avoidance	1.86	0.93
A3	Staying away from reminders	Avoidance	1.79	0.92
A4	Trying not to think	Avoidance	1.80	0.87
A5	Unprocessed feelings	Avoidance	1.86	0.88
A6	Emotional numbness	Avoidance	2.34	1.17
A7	Wanting to forget	Avoidance	1.88	0.99
A8	Avoiding mention	Avoidance	1.84	0.89
H1	Irritability	Hyperarousal	1.84	0.90
H2	Nervousness/exaggerated startle	Hyperarousal	1.64	0.80
H3	Difficulty falling asleep	Hyperarousal	1.63	0.77
H4	Difficulty concentrating	Hyperarousal	1.71	0.82
H5	Physiological reactions	Hyperarousal	1.60	0.78
H6	Hypervigilance	Hyperarousal	1.89	0.95
D1	Loss of interest	Depression	1.62	0.87
D2	Depressed mood	Depression	1.61	0.84
D3	Sleep problems	Depression	1.67	0.92
D4	Fatigue	Depression	1.71	0.91
D5	Appetite changes	Depression	1.66	0.92
D6	Self-deprecation	Depression	1.61	0.88
D7	Difficulty concentrating	Depression	1.64	0.91
D8	Psychomotor changes	Depression	1.53	0.86
D9	Suicidal ideation	Depression	1.34	0.77
–	IES-R Total Score	Overall	40.99	14.22
–	PHQ-9 Total Score	Overall	14.38	6.16

### 3.2 Symptom network structure

**Figure 1:**
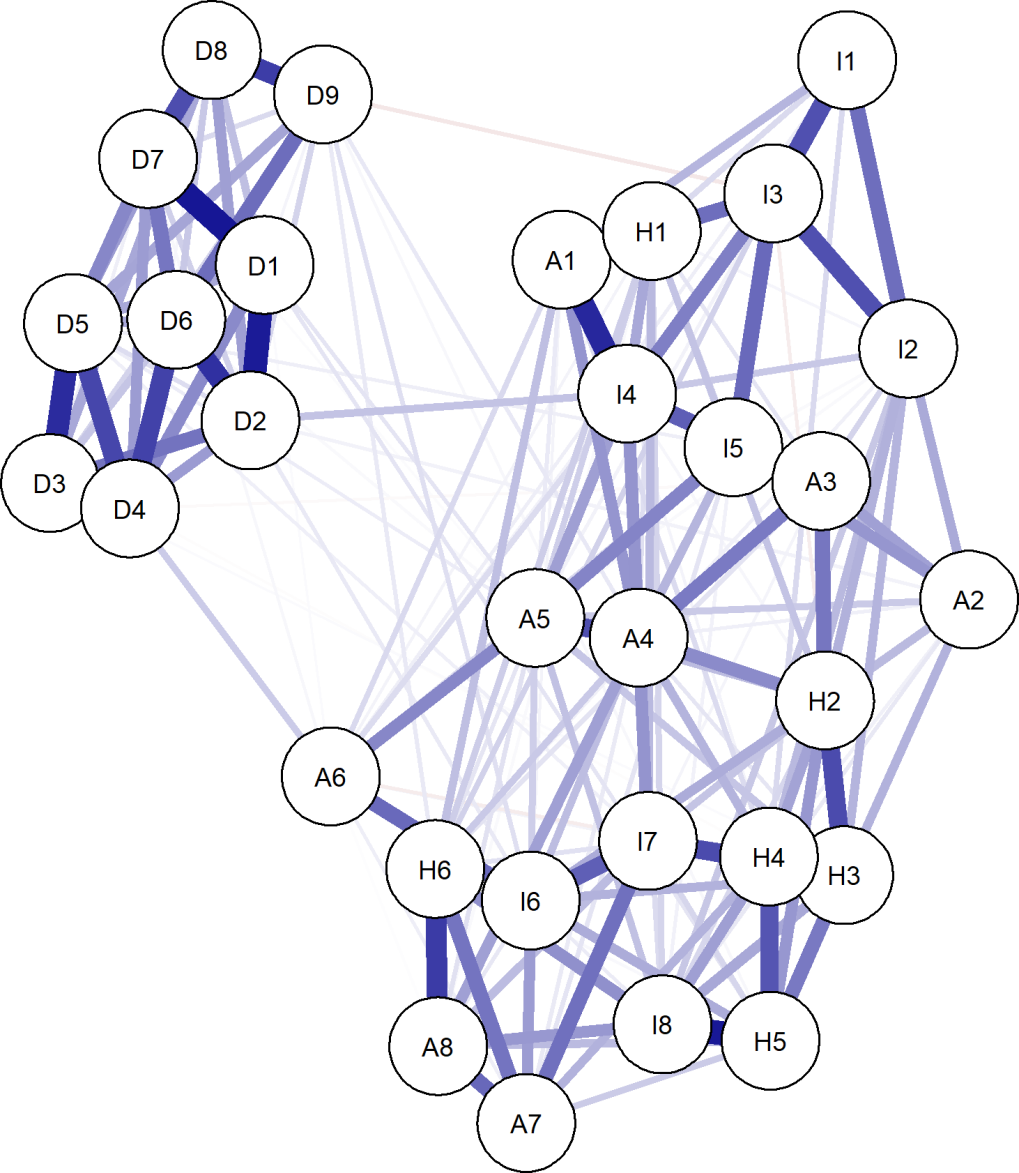
IES-R × PHQ-9 symptom network structure. Nodes represent individual symptoms from the IES-R and PHQ-9. Edges represent partial correlation coefficients between symptoms after regularizing with the graphical LASSO. Blue edges indicate positive correlations, while red edges indicate negative correlations. The thickness of the edges represents the strength of the association. The layout is based on the Fruchterman–Reingold algorithm, where more strongly connected nodes are placed closer together. Nodes are coded by their respective theoretical dimensions: Intrusion, Avoidance, Hyperarousal, and Depression.

**Figure 1** presents the symptom network structure of IES-R × PHQ-9 among college students. The network revealed extensive connections among the 31 symptom nodes, with symptoms belonging to the same dimension tending to cluster together, forming relatively dense groupings. The three subdimensions of post-traumatic stress (intrusion, avoidance, and hyperarousal) exhibited rich intra-dimensional connections, and the depressive symptom cluster also formed a relatively independent community. Notably, cross-diagnostic connections were also significantly present, particularly between the hyperarousal dimension and the depression dimension, which demonstrated considerable cross-community connectivity. This suggests that post-traumatic stress and depression exhibit complex mutual permeation at the symptom level.

Regarding node centrality, I1 (triggered re-experiencing, EI = −2.43), A6 (emotional numbness, EI = −2.31), and A2 (derealization, EI = −1.97) demonstrated the lowest standardized expected influence (Z-scores), suggesting that these symptoms occupy highly peripheral positions within the network, potentially reflecting the unique characteristics of dissociative components in trauma responses.

### 3.3 Central symptom analysis

**Figure 2:**
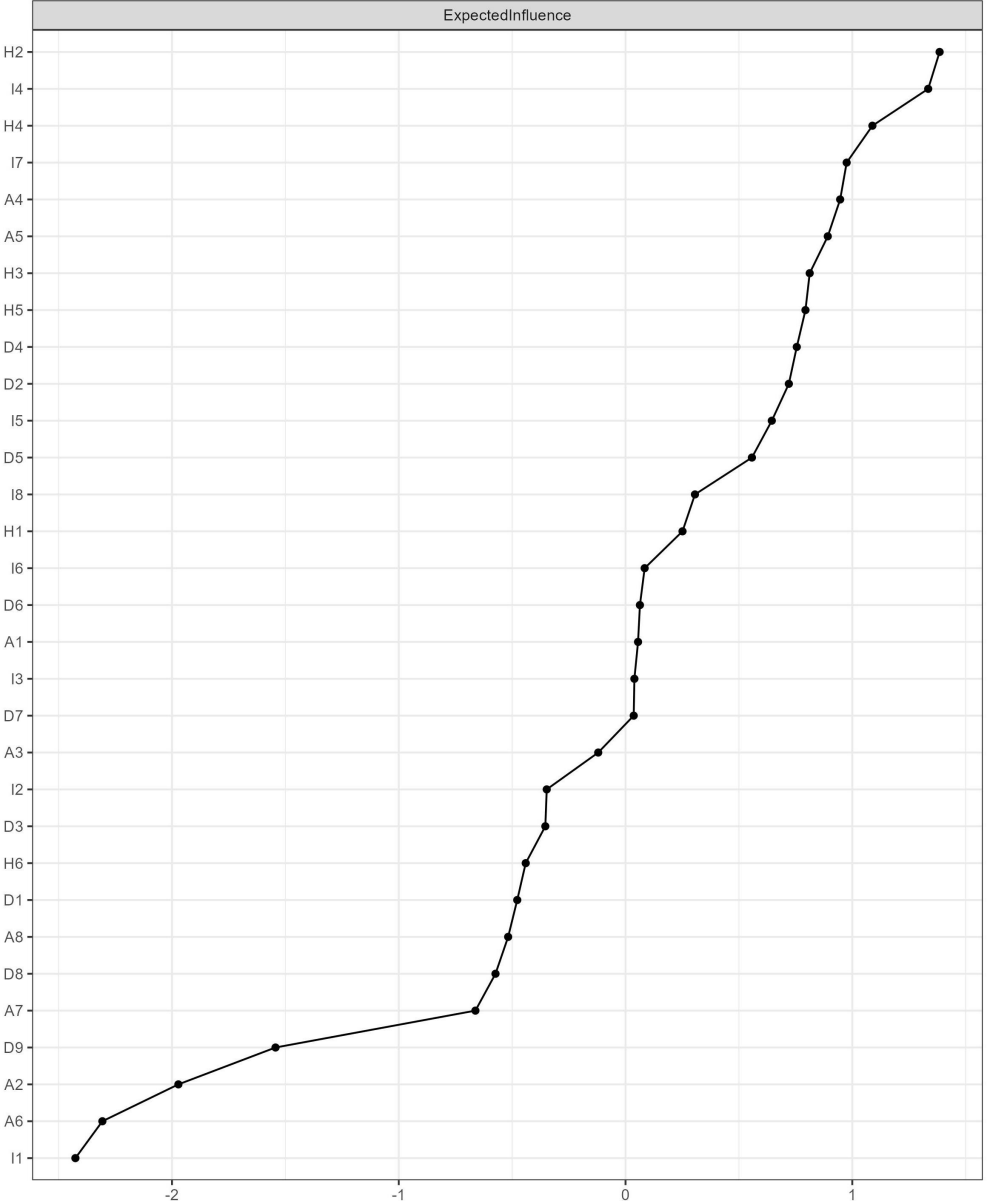
Symptom centrality index (Expected Influence). Expected Influence (EI) represents the sum of the weights of edges connected to a specific node, accounting for the sign of the connections. Higher values indicate greater overall influence or centrality of the symptom within the network. Symptoms are ranked from highest to lowest EI.

**Figure 2** and **Table 3** present the Expected Influence (EI) results for the 31 symptoms. The five symptoms with the highest expected influence were, in descending order: H2 (nervousness/exaggerated startle, EI = 1.38), I4 (involuntary recall, EI = 1.33), H4 (difficulty concentrating, EI = 1.09), I7 (intense emotional fluctuations, EI = 0.98), and A4 (trying not to think, EI = 0.95).

**Table 3:** Symptom Expected Influence rankings.

Rank	Node	Symptom description	Dimension	EI
1	H2	Nervousness/exaggerated startle	Hyperarousal	1.38
2	I4	Involuntary recall	Intrusion	1.33
3	H4	Difficulty concentrating	Hyperarousal	1.09
4	I7	Intense emotional fluctuations	Intrusion	0.98
5	A4	Trying not to think	Avoidance	0.95
6	A5	Unprocessed feelings	Avoidance	0.89
7	H3	Difficulty falling asleep	Hyperarousal	0.81
8	H5	Physiological reactions	Hyperarousal	0.79
9	D4	Fatigue	Depression	0.76
10	D2	Depressed mood	Depression	0.72
…	…	…	…	…
29	A2	Derealization	Avoidance	−1.97
30	A6	Emotional numbness	Avoidance	−2.31
31	I1	Triggered re-experiencing	Intrusion	−2.43

EI = Expected Influence; values are unbounded and may legitimately exceed ±1.0.

The central symptoms were primarily distributed across the hyperarousal and intrusion dimensions. The hyperarousal dimension contributed two of the top five (H2 and H4), the intrusion dimension also contributed two (I4 and I7), and the avoidance dimension contributed one (A4). Notably, the highest-ranked depressive symptom, D4 (fatigue), ranked only ninth, and D2 (depressed mood) ranked tenth, indicating that within this cross-diagnostic network, post-traumatic stress symptoms generally demonstrated higher network centrality, while depressive symptoms more frequently occupied peripheral positions.

### 3.4 Bridge symptom analysis

**Figure 3:**
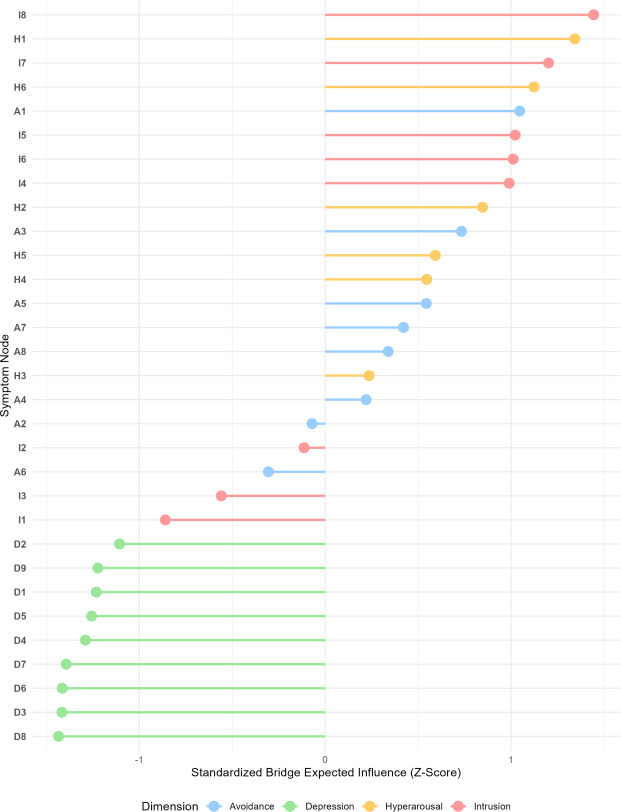
Bridge symptom centrality. Bridge Expected Influence quantifies the extent to which a symptom connects different symptom clusters (Intrusion, Avoidance, Hyperarousal, and Depression). Higher Z-scores indicate a more prominent role as a ‘bridge’ linking post-traumatic stress and depressive domains. Colors of the dots correspond to the four dimensions of the nodes.

**Figure 3** and **Table 4** present the results of the Bridge Expected Influence (Bridge EI) analysis. The five symptoms with the highest bridge centrality were, in descending order: I8 (trauma-related dreams, raw Bridge EI = 0.88, Z = 1.45), H1 (irritability, raw Bridge EI = 0.85, Z = 1.34), I7 (intense emotional fluctuations, raw Bridge EI = 0.80, Z = 1.20), H6 (hypervigilance, raw Bridge EI = 0.78, Z = 1.12), and A1 (avoiding distress, raw Bridge EI = 0.76, Z = 1.05).

**Table 4:** Bridge symptom rankings (top 5).

Rank	Node	Symptom description	Dimension	Bridge EI (raw)	Z-score
1	I8	Trauma-related dreams	Intrusion	0.88	1.45
2	H1	Irritability	Hyperarousal	0.85	1.34
3	I7	Intense emotional fluctuations	Intrusion	0.80	1.20
4	H6	Hypervigilance	Hyperarousal	0.78	1.12
5	A1	Avoiding distress	Avoidance	0.76	1.05

Bridge symptoms were distributed across all three subdimensions of post-traumatic stress, with the intrusion dimension contributing the two highest-ranking bridge symptoms (I8 and I7), the hyperarousal dimension contributing two (H1 and H6), and the avoidance dimension contributing one (A1). This indicates that cross-diagnostic connectivity between post-traumatic stress and depression involves multiple pathways, with intrusion and hyperarousal symptoms serving as the primary bridges.

To more extensively present the Bridge Expected Influence (BEI) analysis and clarify the patterns of symptom co-occurrence, we examined the specific cross-community edge weights. Because the network was classified into four distinct communities (Intrusion, Avoidance, Hyperarousal, and Depression), high BEI values among PTSD symptoms primarily reflected their strong role in consolidating the post-traumatic stress response across its sub-dimensions. Specifically, the highest-ranking bridge symptom, I8 (trauma-related dreams), strongly bridged Intrusion with Hyperarousal (via connections to H5, weight = 0.24; and H6, weight = 0.12) and Avoidance (via A8, weight = 0.11). Similarly, H1 (irritability) served as a critical hub connecting Hyperarousal to Avoidance (via A1, weight = 0.25) and Intrusion (via I3, weight = 0.15). While these hub symptoms act as primary internal bridges that maintain and strongly associate with the trauma response cluster, direct symptom-to-symptom association with the Depressive domain was observed through more specific pathways. For instance, I4 (involuntary recall) showed the strongest direct connection to the depression domain through D2 (depressed mood, weight = 0.06), and A6 (emotional numbness) directly connected to D4 (fatigue, weight = 0.05). This suggests a complex network architecture where post-traumatic stress operates as a tightly coupled symptom cluster that collectively, rather than through single massive edges, interconnects with depressive symptoms.

### 3.5 Overlap between central and bridge symptoms

**Table 5** presents a comprehensive comparison of the central and bridge symptom rankings. I7 (intense emotional fluctuations) appeared simultaneously at rank 4 among central symptoms and rank 3 among bridge symptoms, indicating that this symptom possesses the dual characteristics of “high network influence” and “high cross-dimensional bridging capacity.”.

**Table 5:** Comparison of central and bridge symptom rankings.

Node	Symptom description	Dimension	EI rank	Bridge EI rank	Dual role
H2	Nervousness/exaggerated startle	Hyperarousal	1	—	
I4	Involuntary recall	Intrusion	2	—	
H4	Difficulty concentrating	Hyperarousal	3	—	
I7	Intense emotional fluctuations	Intrusion	4	3	✓
A4	Trying not to think	Avoidance	5	—	
I8	Trauma-related dreams	Intrusion	—	1	
H1	Irritability	Hyperarousal	—	2	
H6	Hypervigilance	Hyperarousal	—	4	
A1	Avoiding distress	Avoidance	—	5	

### 3.6 Network stability and accuracy

Bootstrap test results indicated that the network demonstrated good stability and accuracy. Nonparametric bootstrap (1,000 resamples) showed that the 95% confidence intervals for most edge weights were relatively narrow, indicating high accuracy of the network structure. Case-dropping bootstrap results indicated that the CS-coefficient for Expected Influence (EI) reached 0.75, and the CS-coefficient for Bridge Expected Influence (Bridge EI) also reached 0.75, both substantially exceeding the recommended threshold of 0.50, indicating that the rank ordering of both centrality indices remained stable even when a large proportion of cases were randomly removed. Edge weight difference tests and centrality difference tests further confirmed the robustness of the key findings.

### 3.7 Network comparison test

**Figure 4:**
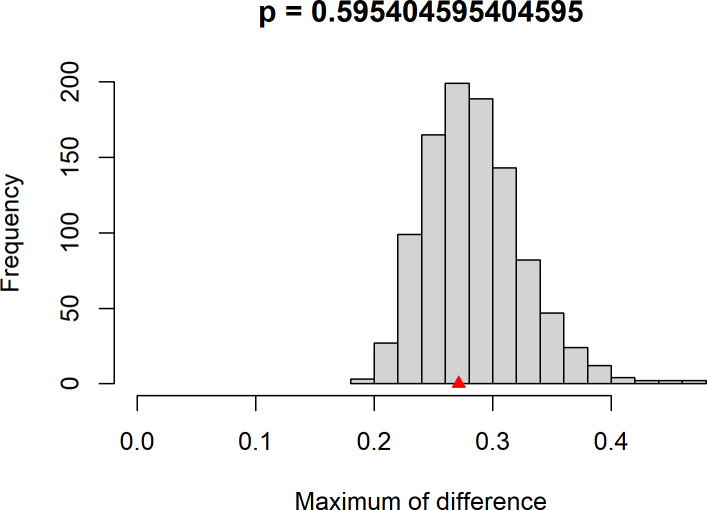
Network structure invariance test. The figure displays the permutation distribution (1,000 iterations) of the maximum difference in edge weights (M) between the high-risk and low-risk PTSD subgroups. The dotted line represents the observed test statistic (M = 0.271), which is not statistically significant (p = 0.595), indicating stable network topology across severity levels.

**Figure 5:**
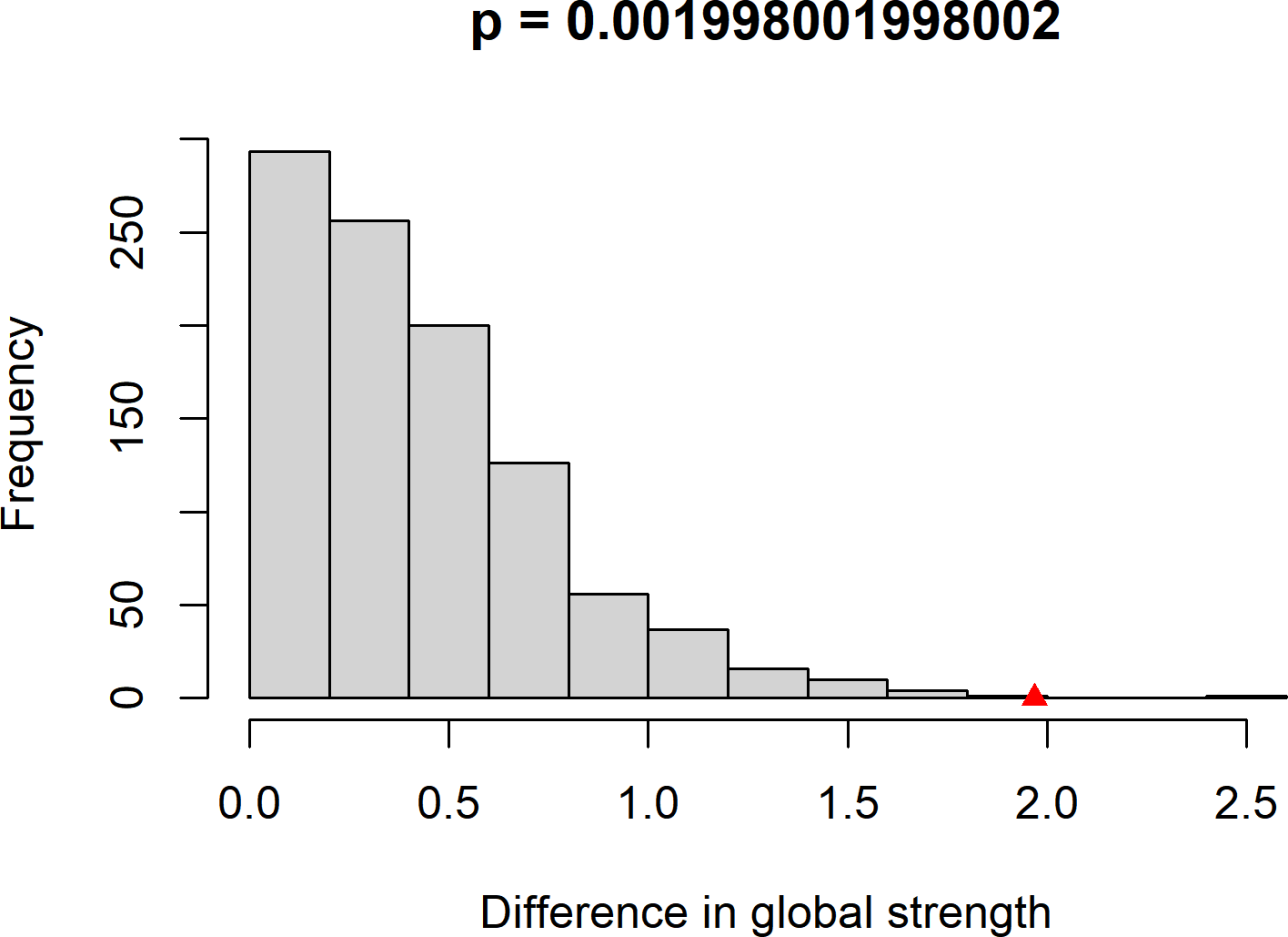
Global strength invariance test. The figure displays the permutation distribution (1,000 iterations) of the difference in global network strength (S) between the high-risk and low-risk PTSD subgroups. The dotted line represents the observed test statistic (S = 1.969), which falls outside the significance threshold (p = 0.002), indicating that the symptom network is significantly more tightly connected in the high-risk group.

To directly evaluate whether the identified network structure is relevant to pathological states, we conducted a Network Comparison Test (NCT) comparing subgroups stratified by the equivalent DSM-5 clinical cutoff for probable PTSD. The sample was split into a high-risk group (IES-R≥47; n = 143) and a low-risk group (IES-R<47; n = 358). The NCT was conducted with 1,000 permutations. Results indicated no significant difference in the overall network structure between the two groups (M = 0.271, p = 0.595; **Figure 4**), suggesting that the fundamental topology, including the prominence of central and bridge symptoms identified in the full sample, remains invariant across severity levels. However, the global network strength was significantly higher in the high-risk group (12.55 vs. 10.58; S = 1.969, p = 0.002; **Figure 5**). This demonstrates that while the core symptom architecture is stable, symptom interconnections become significantly more intensified and tightly coupled in individuals experiencing clinical-level distress.

## 4 Discussion

The present study employed network analysis to systematically explore the cross-diagnostic network structure of post-traumatic stress symptoms (IES-R) and depressive symptoms (PHQ-9) among Chinese college students, successfully identifying central symptoms and bridge symptoms. The following sections discuss the overall network characteristics, clinical significance of central symptoms, bridge symptoms and comorbidity mechanisms, and intervention implications.

### 4.1 Overall characteristics of the symptom network

The IES-R × PHQ-9 cross-diagnostic network constructed in this study exhibited a topological structure dominated by positive connections while also containing several notable negative connections, consistent with the core hypothesis of network theory regarding mutual activation among psychological symptoms (13). The connection strength between symptoms within the same dimension was generally higher than that between cross-dimensional connections, reflecting the reasonableness of both the three-factor structure of the IES-R and the unidimensional structure of the PHQ-9. However, the widespread presence of cross-diagnostic connections—particularly between hyperarousal symptoms and depressive symptoms—suggests that post-traumatic stress and depression are not mutually independent systems but rather permeate and influence each other through specific symptom nodes. Notably, I1 (triggered re-experiencing), A6 (emotional numbness), and A2 (derealization) exhibited the lowest standardized expected influence. This peripheral network position may reflect the unique characteristics of dissociative components in trauma responses—emotional numbness and derealization represent an “emotional shutdown” state that operates relatively independently and is less centrally engaged in the mutual activation cycle of other active symptoms (27). This finding aligns with the clinical observation of dissociative symptoms (e.g., depersonalization and derealization), which are classified as a specific dissociative subtype in the DSM-5. These symptoms may reflect a defensive regulatory mechanism against excessive arousal. Concurrently, the impact of severe and prolonged trauma has also been recognized in the ICD-11 framework, which introduces the distinct diagnosis of Complex PTSD (CPTSD) (28).

Furthermore, the Network Comparison Test (NCT) results provided robust evidence regarding the clinical relevance of this structure. The finding that global network strength significantly intensified in the high-risk PTSD group, while the overall structural topology remained stable, aligns perfectly with the core tenets of the network theory of mental disorders (13). It suggests that the transition from everyday subclinical distress to pathological states is characterized not by a complete rewiring of symptoms, but by a tighter, self-sustaining coupling of the existing network. Consequently, targeting the central and bridge symptoms identified in this baseline network holds significant promise for early prevention before the symptoms consolidate into severe comorbidities.

### 4.2 Clinical significance of central symptoms

This study found that H2 (nervousness/exaggerated startle response) was the node with the highest expected influence in the entire cross-diagnostic network (EI = 1.38). The exaggerated startle response is a hallmark feature of the PTSD hyperarousal symptom cluster, reflecting post-trauma hypersensitivity to threat stimuli and a sustained state of vigilance (29). The high centrality of this symptom may be consistent with its potential capacity to co-occur with other symptoms through multiple channels, though experimental or longitudinal evidence would be needed to confirm any associative role: on the one hand, through sustained physiological hyperarousal covarying with other post-traumatic stress symptoms (such as difficulty falling asleep and difficulty concentrating), and on the other hand, indirectly linked to greater depressive severity through impairment of daily functioning. This finding is consistent with the network analysis results reported by Yuan et al. (30) in a sample of Chinese firefighters, which similarly identified the exaggerated startle response as one of the most central symptoms in the PTSD network.

The second-ranked symptom, I4 (involuntary recall, EI = 1.33), represents a core intrusion dimension symptom. Involuntary recall is one of the most characteristic symptoms of PTSD, involving the uncontrollable and recurrent emergence of traumatic memories in consciousness (31). From a cognitive-theoretical perspective, involuntary recall reflects the failure to adequately integrate traumatic memories into the autobiographical memory system, resulting in fragmented sensory and emotional memories persistently intruding into current consciousness (32). Its high centrality suggests that involuntary recall may be closely interlinked with a cluster of avoidance behavior and emotional distress, potentially reflecting a strong co-occurrence with trauma-related negative emotions and physiological arousal.

H4 (difficulty concentrating, EI = 1.09), as the third-ranked central symptom, is particularly noteworthy because attentional difficulty appears in both the IES-R (H4) and the PHQ-9 (D7). Attention is a limited cognitive resource, and both post-traumatic stress and depression can impair attentional function through different mechanisms—the former through hypervigilance causing attention to be captured by threat-related stimuli, and the latter through rumination and cognitive inhibition consuming attentional resources (33). The high centrality of attentional symptoms in the cross-diagnostic network is consistent with a growing body of research suggesting that cognitive function impairment may represent a shared vulnerability factor between post-traumatic stress and depression (34).

Notably, depressive symptoms exhibited relatively low centrality in the overall network, with the highest-ranked, D4 (fatigue), ranking only ninth. This pattern suggests that in the network dynamics of post-traumatic stress–depression comorbidity, post-traumatic stress symptoms—particularly hyperarousal and intrusion—may exhibit higher global centrality, while depressive symptoms more frequently operate as peripheral positions within the concurrent network. This inference is logically consistent with findings by Cheng et al. (35), who demonstrated through cross-lagged analysis that PTSD symptoms exhibited stronger temporal associations with future depressive symptoms, rather than the reverse direction.

### 4.3 Bridge symptoms and comorbidity mechanisms

Bridge symptom analysis constitutes one of the core findings of this study. I8 (trauma-related dreams) exhibited the highest Bridge Expected Influence (Bridge EI = 0.88, Z = 1.45), indicating that this symptom shows the strongest cross-diagnostic network connections, suggesting it may serve as an important node linking post-traumatic stress and depressive symptoms, though the direction of this association cannot be determined from the current cross-sectional design. As a specialized manifestation of intrusion symptoms, trauma-related dreams are theoretically associated with depressive symptoms through multiple pathways: nightmares are closely linked to deterioration in sleep quality, and sleep disturbance is an important risk factor for the development of depression (36); recurrent trauma-related dream experiences can also elicit learned helplessness and fear of sleep, further exacerbating low mood and functional impairment. Geng et al. (37) found that sleep-related symptoms served as an important associated pathway between PTSD and depression, and the present study further validated this mechanism among college students.

H1 (irritability, Bridge EI = 0.85, Z = 1.34), as the second-ranked bridge symptom, also warrants attention. Irritability represents a nonspecific state of emotion regulation difficulty situated at the intersection of PTSD and depression: it is both a direct manifestation of post-traumatic hyperarousal and can increase depression risk through interpersonal conflicts leading to the loss of social support (38). Wei et al. (20) also reported similar results in a nursing staff sample, finding that restlessness and agitation served as bridge symptoms connecting PTSD and depression.

I7 (intense emotional fluctuations, Bridge EI = 0.80) simultaneously ranked as both a central symptom (EI rank 4) and a bridge symptom (Bridge EI rank 3), and its dual role is particularly noteworthy. Emotional fluctuations reflect impaired emotion regulation functioning, which plays a central role in both post-traumatic stress and depression (39). Emotional fluctuations may relate to comorbidity through the following pathway: intense emotional experiences triggered by trauma, failure of emotion regulation, negative self-evaluation, and concurrently, depressed mood. This associated pathway is highly consistent with existing emotion regulation theory, which posits that emotion dysregulation is a transdiagnostic factor underlying multiple forms of psychopathology (40).

Taken together, bridge symptoms predominantly originated from the post-traumatic stress dimensions (rather than the depression dimension) and were connected primarily through “emotion–physiological” channels (dreams → sleep, irritability → interpersonal relations, emotional fluctuations → emotion regulation). This suggests that emotion regulation and sleep may constitute the two most critical cross-diagnostic bridging mechanisms in post-traumatic stress–depression comorbidity.

### 4.4 Theoretical and clinical significance of the central–bridge overlap

I7 (intense emotional fluctuations) simultaneously exhibited high centrality and high bridge centrality, and this overlapping pattern carries important theoretical and practical implications. From a network theory perspective, this symptom not only possesses strong associative strength within the overall network but also plays the most important connecting role between post-traumatic stress and depression, and may function as a hub symptom that is centrally positioned in the comorbid network, warranting further investigation into its role in the development and maintenance of comorbidity. From an intervention perspective, this implies that targeted treatment addressing emotional fluctuations may yield dual benefits: reducing the overall severity of post-traumatic stress symptoms while simultaneously decreasing the risk of co-occurrence in the depressive domain.

### 4.5 Intervention implications

Because the present network captures symptom interactions at a subclinical stage, these findings have important implications for early mental health prevention. Identifying and targeting key central and bridge symptoms (e.g., H2, I8) before they consolidate into severe clinical thresholds can provide university counseling centers with a critical window to disrupt the trajectory toward formal PTSD-depression comorbidity. First, interventions targeting central symptoms are likely to produce the greatest overall network symptom reduction. For H2 (nervousness/exaggerated startle response), exposure therapy and Cognitive Processing Therapy (CPT) can effectively reduce post-traumatic hyperarousal levels. For I4 (involuntary recall), imagery rescripting techniques can help individuals reprocess fragmented traumatic memories and reduce involuntary intrusions. Second, interventions targeting bridge symptoms can effectively block cross-diagnostic co-occurrence. For I8 (trauma-related dreams), Imagery Rehearsal Therapy (IRT) is a first-line psychological intervention for post-traumatic nightmares that can simultaneously improve sleep quality and reduce post-traumatic stress symptoms. For H1 (irritability), emotion regulation skills training—such as distress tolerance skills and mindfulness techniques from Dialectical Behavior Therapy (DBT)—can directly target emotional reactivity. Third, the present findings support the application of transdiagnostic intervention strategies. The dual role of emotional fluctuations (I7) as both a central and bridge symptom suggests that transdiagnostic treatment protocols centered on emotion regulation—such as the Unified Protocol—may offer unique advantages in addressing PTSD–depression comorbidity. Finally, for university mental health practitioners, it is recommended that particular attention be paid to the central and bridge symptoms identified in this study during screening and assessment. When students report significant exaggerated startle responses, trauma-related nightmares, or difficulties with emotion regulation, clinicians should be alert to the risk of PTSD–depression comorbidity development and implement targeted interventions as early as possible.

### 4.6 Limitations and future directions

Several limitations of the present study should be noted. First, the cross-sectional design precludes causal inference or determination of temporal ordering among symptoms; the edges in the network reflect only conditional dependency relationships rather than causal effects. Future research could employ Ecological Momentary Assessment (EMA) to collect intensive time-series data and construct temporal networks to reveal dynamic causal relationships among symptoms. Second, this study employed a convenience sample of undergraduate students recruited via snowball sampling across four Chinese provinces, which may introduce self-selection bias and limits generalizability to other populations. While college students constitute a theoretically and clinically relevant population given their unique stress exposures, the network structure identified here may not directly apply to clinical samples, older adults, or populations with chronic or severe trauma histories. Future studies should seek to replicate these findings in clinically referred student populations and population-representative community samples. Third, the IES-R is based on the DSM-IV three-factor model of PTSD (intrusion, avoidance, and hyperarousal) and does not encompass the fourth dimension added in the DSM-5—Negative Alterations in Cognitions and Mood (NACM). Future studies may consider using the PCL-5 to achieve a more comprehensive assessment of PTSD symptoms. Fourth, although the ratio of sample size (N = 501) to the number of nodes (31) was approximately 16:1, meeting the requirements for network analysis, and the CS-coefficient was excellent, a larger sample size would further improve the precision of edge weight estimates. Moreover, while the IES-R instructions prompted participants to focus on their most impactful recent stressor, we did not systematically screen for, classify, or control for the specific types and severity of the traumatic events experienced. Different trauma types (e.g., interpersonal vs. non-interpersonal trauma) may give rise to different network structure patterns, and future research should compare network differences across specific trauma categories. Finally, symptoms of post-traumatic stress and depression are influenced not only by symptom-to-symptom associations but also by broader environmental factors, such as trauma characteristics, cumulative stress, and social context. Recent research highlights the critical role of environmental exposures, the exposome, in shaping psychiatric symptoms and underlying neurobiology (41). Specifically, diverse environmental factors, ranging from urbanization to early life adversity, have been identified as critical modifiable risk factors that significantly influence depression risk and progression through interconnected biological pathways (42). Because such broad environmental factors were not comprehensively measured in the present study, the observed network may partly reflect these unmeasured environmental influences. Future network studies should integrate multi-level environmental assessments to better contextualize symptom interactions.

## 5 Conclusion

The present study employed network analysis to explore the cross-diagnostic network structure of post-traumatic stress symptoms and depressive symptoms among Chinese college students, systematically identifying central and bridge symptoms. The findings revealed that: (1) nervousness/exaggerated startle response (H2), involuntary recall (I4), and difficulty concentrating (H4) were the central symptoms with the highest expected influence in the network, primarily distributed across the hyperarousal and intrusion dimensions, suggesting that post-traumatic stress symptoms exhibit high centrality within the cross-diagnostic network; (2) trauma-related dreams (I8), irritability (H1), and intense emotional fluctuations (I7) were key bridge symptoms connecting post-traumatic stress and depression, achieving cross-diagnostic associations primarily through sleep and emotion regulation channels; (3) emotional fluctuations (I7) simultaneously exhibited high centrality and high bridge centrality and should be considered a priority intervention target; (4) the network demonstrated excellent stability (CS = 0.75), supporting the robustness of the conclusions; and (5) the Network Comparison Test (NCT) validated the pathological relevance of this network, demonstrating that while the core topology remains stable, symptom interconnections significantly intensify at clinical thresholds. These findings provide empirical evidence for early, precision-oriented mental health interventions among college students. By targeting highly central and bridging symptoms in subclinical stages, university psychological services may more effectively disrupt the consolidation of everyday distress into formal clinical comorbidities.

## Data Availability

The raw data supporting the conclusions of this article will be made available by the authors, without undue reservation.
